# Expression of Concern: Analyzing intelligent tourism development and public services based on a fuzzy genetic hybrid system to promote environmental and cultural values

**DOI:** 10.1371/journal.pone.0362639

**Published:** 2026-11-12

**Authors:** The PLOS One Editors

After this article [1] was published, the following concerns were noted:

- The article cited as Reference 5 was retracted before [1] was published.
- The articles cited as References 1, 12, and 34 were retracted after [1] was published.

In addition, the *PLOS One* Editors have concerns about the article’s peer review.

In light of the cumulative issues, the *PLOS One* Editors issue this Expression of Concern. Readers are advised to interpret the article [1] with caution.
