# Supplementary material for: Analyzing intelligent tourism development and public services based on a fuzzy genetic hybrid system to promote environmental and cultural values
Source: PLoS One. 2024 Jul 9;19(7):e0306718. doi: 10.1371/journal.pone.0306718 (PMC11233016; doi:10.1371/journal.pone.0306718)
Supplement: Attachment — Submitted filename: Response to reviewers.docx [file pone.0306718.s001.docx]

Journal Requirements:
Upon resubmission, please provide the following:
* The name of the colleague or the details of the professional service that edited your manuscript
* A copy of your manuscript showing your changes by either highlighting them or using track changes (uploaded as a supporting information file)
* A clean copy of the edited manuscript (uploaded as the new manuscript file)

1. We therefore request that you revise the text to fix the grammatical errors and improve the overall readability of the text. We suggest you have a fluent English-language speaker thoroughly copyedit your manuscript for language usage, spelling, and grammar. If you do not know anyone who can do this, you may wish to consider employing a professional scientific editing service.

**Ans:** Proofreading is done. Grammatical errors have been rectified.

2. Please review your reference list to ensure that it is complete and correct. If you have cited papers that have been retracted, please include the rationale for doing so in the manuscript text, or remove these references and replace them with relevant current references. Any changes to the reference list should be mentioned in the rebuttal letter that accompanies your revised manuscript. If you need to cite a retracted article, indicate the article’s retracted status in the References list and also include a citation and full reference for the retraction notice.

**Ans:** The retracted article has not been cited in this manuscript.
